# Knowledge on mucormycosis among nursing Indian students in the state of Gujarat

**DOI:** 10.6026/973206300191003

**Published:** 2023-10-31

**Authors:** R Ambiha, R Gopal, N Siva Subramanian, Girishkumar Patel Bansi, Grishbhai Patel Dhenu, Baldevbhai Patel Dharmik, Vikrambhai Patel Disha, Alpeshkumar Patel Heli, Nareshbhai Patel Janu

**Affiliations:** 1 Nootan College of Nursing, Sankalchand Patel University, Visnagar, Gujarat-384315, India

**Keywords:** Knowledge, mucormycosis, nursing students, covid-19, black fungus

## Abstract

The disorder known as angio-invasive mucormycosis is characterized by tissue necrosis and infarction. The Mucorales order of saprophytic fungi is responsible for its development. It is unclear how widespread mucormycosis is in India due to a lack of population-based investigations. Diabetes mellitus is the risk factor that occurs the most frequently, followed by solid organ transplant and hematological cancer. The present study has been carried out to assess the knowledge regarding mucormycosis among nursing students from Nootan College of Nursing, Visnagar, Gujarat. For this we have selected 100 students by using the probability sampling technique. Structured questions were used to assess the knowledge of nursing students regarding mucormycosis. The Score was categorized as poor, average and good. The results show that 45(45%) of the nursing students having poor knowledge, 35(35%) of them were having average knowledge, 20(20%) of them were having good knowledge. There is an association between gender, program and their level of knowledge. Most of the students having poor knowledge regarding mucormycosis and we need to create awareness regarding mucormycosis to Nursing students.

## Background:

A group of filamentous moulds belonging to the order Mucorales are responsible for the infection known as mucormycosis. Food contamination, spore inhalation into the nose or lungs, or inoculation into broken skin or wounds can all cause infections. [1]. Mucormycosis is an uncommon, difficult-to-diagnose illness with a high fatality rate. The disease often progresses quickly and diagnosis is frequently delayed. The need for immediate surgical and medical intervention is vital [2]. In India, the estimated prevalence of Mucormycosis is almost 70 times higher than it is globally [3]. Mucormycosis may have become epidemic during the COVID-19 pandemic, the name was well-known long before that time. A novel coronavirus outbreak (SARS-CoV-2) that infected millions of people worldwide occurred in the year 2020. Mucormycosis is one of the prominent consequences that have been linked to this illness. It is an opportunistic infection brought on by members of the Order Mucorales, which are found all over the world and have long been documented as a laboratory contaminant [4].

Over the past few decades, India has recorded an increasing number of cases of mucormycosis. Indian mucormycosis has a few distinctive characteristics. The most common symptom is a rhino-orbito-cerebral presentation linked to uncontrolled diabetes. A new clinical entity called isolated renal mucormycosis has been identified. Emerging species in this area include *Apophysomyces elegans*, *Rhizopus homothallicus*, *Mucor irregularis*, and *Thamnostylum Lucknowense*. Uncommon pathogens like these are also being documented [5]. With the increase in incidence, the discovery of new causal agents, and the emergence of a sensitive population, there has been a change in the epidemiology of mucormycosis in recent years. The increase has been noticed by people all across the world, but it is most noticeable in Asia. In Asia, diabetes mellitus still outweighs all other risk factors, but post-tuberculosis and chronic renal failure has arisen as new risk groups [6]. Numerous risk factors, such as type 2 diabetes, ketoacidosis, haematological malignancies, organ transplant recipients, and chemotherapy patients, can lead to the development of mucormycosis. Rhizopus is discovered to be the most prevalent etiological agent among the several etiological agents, and rhino-cerebral is the most prevalent clinical presentation [4]. Normal immune function was hampered by COVID-19 infection and subsequent treatment with steroids and immune modulatory drugs. A further infection known as Black Fungus (Mucormycosis) emerged as a result of the immune dysfunction that followed. However, with the right information, a knowledgeable mindset, and deliberate preventive behavior, the spread of black mold can be reduced [7]. Due to a dearth of population-based studies, it is uncertain how common mucormycosis is in India. In India, the estimated prevalence of mucormycosis is almost 70 times higher than it is globally. Important issues with mucormycosis in India include the growth in cases, the appearance of new risk factors and causative agents, and the difficulties in managing the illness [3].

## Methodology:

This study used a quantitative research approach and a survey research design. A simple random sample technique was used to choose 100 nursing students who satisfied the study's inclusion criteria from Nootan College of Nursing in Visnagar. The study tool is divided into two parts. Demographic variables were assessed by structured interview it contains Age (In year), Programme, Gender, Knowledge regarding mucormycosis and Source of information regarding mucormycosis and Level of knowledge was assessed by using self-structured questionnaires. It compromised 25 multiple choice questions in a single correct answer. Every correct answer was awarded one score and every incorrect was awarded as 0 score. The maximum score on knowledge was 25 and minimum score was zero. Formal administrative permission to conduct study was obtained from Nootan College of Nursing, Visnagar. The nature, purpose and aim of the study were explained to the nursing students. Self-Structured tool was given to selected samples. The average time taken by nursing students to fill the questionnaire was 10-15 minutes.

## Results:

Table 1 shows the frequency & percentage distribution of the sample according to the demographic variables. Most of the nursing students 81 (81%) comes from 17-22 years of age, 55 (55%) of the Nursing students studying B.Sc Nursing, 71 (71%) of them were Females, 68 (68%) of them don't have previous knowledge regarding mucormycosis and 48 (48%) of the Nursing students got information regarding mucormycosis from Mass media.

**Figure 1:**
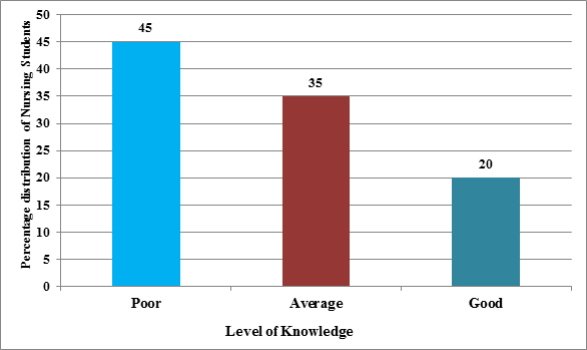
Percentage distribution according to the level of knowledge among nursing students.

Figure 1 displays the frequency and percentage distribution of the samples. Given that 45 (45%) of the nursing students had poor knowledge, 35 (35%) had average knowledge and 20 (20%) had good knowledge, it appears that education intervention in the area of mucormycosis is necessary for the nursing students.

Table 2 depicts that the association between level of knowledge regarding mucormycosis among nursing students and their selected demographic variables. Demographic variables, Programme and Gender had shown statistically significant association between levels of knowledge regarding mucormycosis among nursing students at P=0.05 level of significant. The other demographic variables like age, knowledge regarding mucormycosis and source of information regarding mucormycosis had not shown statistically significant association between levels of knowledge regarding mucormycosis among nursing students.

## Discussion:

A prospective study of 38 patients who were diagnosed with mucormycosis in a specialized care center between January 2010 and June 2011 was conducted. The cases' involvement site, underlying illness, isolated fungus species, susceptibility pattern to antifungal medications, and therapeutic results were all examined. 72% of the patients were men, with a mean age of 40-43. Rhino-orbital mucormycosis was the most prevalent manifestation (61.5%), followed by cutaneous signs (31%), gastrointestinal symptoms (5%), and pulmonary (2.5%). The significant risk factor for rhino-orbito-cerebral presentation was diabetes mellitus (56%) (OR = 7.55, P = 0.001) [8].

The estimated incidence of mucormycosis is 0.14 instances per 1000 persons in India, which is around 70 times higher than the expected incidence of mucormycosis worldwide. 82 cases of mucormycosis were discovered out of the 6365 samples that were collected for mycological culture and examination within the designated time period. Out of these, there were 56 male patients and 27 female patients. The most frequent presentations were rhino-orbito-cerebral (37), cutaneous (25), pulmonary (14), oral cavity involvement (4), and gastrointestinal (2) [9]. A total of 222 individuals, or 66%, had some awareness of mucormycosis, while 98/222 participants, or 44%, had no knowledge of mucormycosis despite being admitted to the hospital. More than 40% of them claimed that mainstream media was their main source of information. The possibility that it can happen after COVID-19 infection was known to almost 81% of the responders. Only 25 of them were aware that the primary risk factor was the use of systemic steroids. Of 124 participants, 64 were aware that diabetes is a significant risk factor. A COVID vaccine can prevent mucormycosis, according to 50% of respondents [10]. From India's Northern, Southern, Western, and Eastern areas, a total of 437 respondents were included. Out of these, 210 (48.1%) were from private colleges, while 227 (51.9%) belonged to the government. Females made up over three quarters of the respondents (N=328, 75.1%). According to a classification of participants based on the degree of accurate information, the majority of students (232, 53.1%) had good knowledge regarding mucormycosis and its management [11]. The study had 4573 participants in total. A total of 83% of participants overall knew something about mucormycosis, and 86% understood that this was an emergency. Among the participants, more than 50% were unaware that diabetes raises the risk of mucormycosis [12]. One study from Bangladesh found that the majority of pupils (63.8%) spent close to two hours researching COVID-19 and Black Fungus (Mucormycosis) on electronic and social media. 32.9 percent of the students had low KAP scores, compared to 26% who had good KAP scores. Our findings indicate a substantial relationship between KAP and sex, education, living circumstances, place of residence, and media exposure [6]. In one retrospective analysis, the prevalence of hospitalizations due to mucormycosis was estimated to be 0.12 per 10,000 discharges between January 2005 and June 2014. The average length of stay was 17 days, and 23% of patients were deceased when they were discharged. High readmission rates led to 30 and 37% of patients being readmitted within one and three months, respectively [13].

## Conclusion:

Data shows that the majority of nursing Indian students in the state of Gujarat knew little about mucormycosis, so it is important to create an effective teaching strategy at colleges that includes training.

## Figures and Tables

**Table 1:** Frequency & Percentage distribution of the sample according to the demographic variables

**Sr No.**	**Demographic variables**	**Frequency (N)**	**Percentage (%)**
1	Age (In year)		
	17-22	81	81
	22.1-29	14	14
	29.1-35	5	5
2	Programme		
	B.Sc	55	55
	D.GNM	45	45
3	Gender		
	Male	29	29
	Female	71	71
4	Previous Knowledge regarding mucormycosis.		
	Yes	32	32
	No	68	68
5	Source of information regarding mucormycosis		
	Mass media	48	48
	Self-reading	24	24
	Health personal	17	17
	Academic education	11	11

**Table 2:** Association between level of knowledge regarding mucormycosis among nursing students and their selected demographic variables

**S. No**	**Demographic Variables**	**Level of knowledge**			**Chi-Square x2**	**T-Value**	**Level of Significance**
		Poor	Average	Good			
1	Age (In year)						
	17-22	35	29	17			
	22.1-29	8	4	2	1.4831	9.49	NS
	29.1-35	3	1	1			
2	Programme						
	B.Sc (N)	14	23	18	19.649	5.99	S
	D.GNM	31	10	4			
3	Gender						
	Male	20	7	2	10.4204	5.99	S
	Female	25	26	20			
4	Knowledgeregarding mucormycosis.						
	Yes	17	8	7			
	No	28	27	13	2.1176	5.99	NS
5	Source of information regarding mucormycosis						
	Mass media			13			
	Self-reading	20	15	3			
	Health personal	15	6	3			
	Academic education	7	7		5.5978	12.59	NS
		5	5	1			
S = Significant; NS = Non-Significant
